# Indoor Thermal Environment Long-Term Data Analytics Using IoT Devices in Korean Apartments: A Case Study

**DOI:** 10.3390/ijerph17197334

**Published:** 2020-10-08

**Authors:** Hyunjun Yun, Jinho Yang, Byong Hyoek Lee, Jongcheol Kim, Jong-Ryeul Sohn

**Affiliations:** 1 Department of Health and Safety Convergence Science, Graduate School of Korea University, 73, Goryeodae-ro, Seongbuk-gu, Seoul 02841, Korea; hyunjun0116@korea.ac.kr (H.Y.); iamjinho0@naver.com (J.Y.); 2 The Environment Technology Institute, Coway Co., LTD, 1, Gwanak-ro, Gwanak-gu, Seoul 08826, Korea; byonghyl@coway.co.kr (B.H.L.); jckim@coway.co.kr (J.K.); 3 Department of Public Health Science, Graduate School of Korea University, 73, Goryeodae-ro, Seongbuk-gu, Seoul 02841, Korea

**Keywords:** thermal environment, temperature, relative humidity, IoT-based devices, long-term, personal exposure

## Abstract

IoT-based monitoring devices can transmit real-time and long-term thermal environment data, enabling innovative conversion for the evaluation and management of the indoor thermal environment. However, long-term indoor thermal measurements using IoT-based devices to investigate health effects have rarely been conducted. Using apartments in Seoul as a case study, we conducted long-term monitoring of thermal environmental using IoT-based real-time wireless sensors. We measured the temperature, relative humidity (RH), and CO_2_ in the kitchen, living room, and bedrooms of each household over one year. In addition, in one of the houses, velocity and globe temperatures were measured for multiple summer and autumn seasons. Results of our present study indicated that outdoor temperature is an important influencing factor of indoor thermal environment and indoor RH is a good indicator of residents’ lifestyle. Our findings highlighted the need for temperature management in summer, RH management in winter, and kitchen thermal environment management during summer and tropical nights. This study suggested that IoT devices are a potential approach for evaluating personal exposure to indoor thermal environmental risks. In addition, long-term monitoring and analysis is an efficient approach for analyzing complex indoor thermal environments and is a viable method for application in healthcare.

## 1. Introduction

Thermal stress is a major health risk factor with its implications likely to worsen as global warming intensify the occurrence of extreme weather [1]. Climate change predictions indicate the possibility of increased extreme weather events and unpredictable weather patterns if global climate change continues on the current trajectory [2]. Extreme weather events are known to cause serious public health problems [3]. During the European heatwave of 2003, approximately 2091 deaths were reported in the UK; and according to the World Health Organization (WHO), over 70,000 deaths were reported across Europe, between June and September the same year [4,5,6]. Extreme cold weather and heat waves are known to cause death from acute myocardial infarction (AMI), Dilaveris et al. reported that ambient temperature is an important predictor of AMI mortality [2,7,8]. Awareness of extreme weather implications on health has seen increased interest in research on the relationship between weather and health, with a major focus on the outdoor thermal environment.

People in developed countries spend more than 90% of their time indoors [9]. Therefore, they are more exposed to indoor than outdoor thermal environmental risks [3,10,11]. However, to date, only short-term analysis of indoor thermal environmental health risks for a limited number of residential and public areas exists. Additionally, the use of the existing studies’ findings in the evaluation of health effects is limited by accuracy concerns, resulting from the unstandardized classification of thermal environmental risk exposure [3,12]. 

Indoor thermal environmental has a huge potential to affect health therefore, monitoring of indoor thermal environment has become indispensable [7,13,14,15]. In recent years, the development and dissemination of sensors with improved accuracy has seen great progress and revolutionized the existing situation [16]. Further, IoT-based real-time wireless sensors enable complex measurements of indoor air quality. However, while there have been studies comparing indoor and outdoor thermal environmental for a period of time, studies on long-term on-site monitoring of indoor thermal environmental using IoT devices, and its health effects are minimal. 

Climate changes have the potential to affect indoors thermal environment, therefore, for accuracy and viability, long-term monitoring of thermal environmental, covering different climatic seasons is required. Additionally, the thermal environment in residential spaces is influenced by human activities and differs depending on the purpose (for example, living room, bedroom, and kitchen) and the occupant’s lifestyle [17,18]. Therefore, more studies on individualized spatial thermal monitoring are required. This study aimed at addressing seasonal and individualized spatial indoor thermal environmental risk exposure. Using apartments in Seoul as a case study, we did long-term monitoring of thermal environmental using IoT-based real-time wireless sensors. We analyzed the characteristics and influencing factors of the complex thermal environment. We also suggested an accurate method to measure and analyze indoor thermal environmental data viable for application in the public healthcare system. To the best of our knowledge, this study is the first to attempt the application of long-term indoor thermal environmental data in healthcare.

## 2. Materials and Methods 

### 2.1. Season Classification

This study was conducted in eight apartment buildings in Korea using IoT-based real-time wireless sensors from March 2016 to March 2017. Climatic seasons were grouped and analyzed based on their meteorological characteristics; spring (March to May) and autumn (September to November) have similar meteorological characteristics and were analyzed together, while winter (December to February) and summer (June to August) with different seasonal characteristics were analyzed separately. Additionally, we included tropical night, a meteorological phenomenon that occurs during summer in Korea with temperatures above 25 °C from 6 pm to 6 am. We analyzed tropical nights selected by the Korean Meteorological Administration (KMA) in 2016 [19]. 

### 2.2. Case Study Dwellings

One target facility was selected from each of the eight apartment blocks. Table 1 contains a summary of the characteristics of the eight apartments where the field measurements took place. 10–12 sensors were installed in each apartment. The target facilities had an average size of approximately 93 m^2^ and 2 or 3 bedrooms and were located on various floors, (lower to upper, of high-rise apartment buildings). None of the homes had heating, ventilation, and air conditioning (HVAC) systems, but all had a portable air conditioner.

### 2.3. Indoor Measurements

Wireless IoT devices, adjusted for indoor dry-bulb temperature and indoor relative humidity, were used for long-term measurement of the indoor environmental variables. Table 2 summarizes equipment characteristics and type. The devices were installed in the bedrooms, (2 devices per bedroom), kitchen (2 devices), and living room (2 devices) of each target facility and data were transmitted to the storage server. Measurements were taken at an interval of one minute and were automatically saved to the storage server on an hourly basis. There were approximately 95,550,984 min data, and the total file size was approximately 551,897,468 bytes. During summer and autumn, short-term measurements (eight-day increments) of global temperature and airflow rate were taken from apartment “A”. Special measures were taken to ensure that the measuring instruments did not intrude on the living space; the devices were placed in the center of the room, 1.2 m from the floor and away from any electronic devices that might generate heat, such as laptops, televisions, and monitors. Operative temperature data were recorded 30 min after installation, to ensure the stabilization of the global temperature. Mean radiant temperature (MRT) was calculated from the indoor globe temperature, in accordance with ISO 7726. The indoor globe temperature was measured using a globe with a diameter of 150 mm, in accordance with ISO 7726 [20]. 

### 2.4. Ambient Measurements

For indoor dry-bulb temperature and relative humidity, the IoT device was linked to the KMA weather-service measurement station that was closest to the measurement site and programmed to save data to a remote cloud server every hour [21].

### 2.5. Standard Analysis

The seasonal characteristics of the indoor thermal environment were compared to those of the comfort zone in the American Society of Heating, Refrigerating, and Air-Conditioning Engineers (ASHRAE) Standard 55 [22]. The indoor thermal environment during summer and autumn was analyzed using EN15251. This study shows the operative temperature found for the recommended temperature limits given in EN15251 Annex A for buildings without AC equipment [23]. Based on the procedure provided byEN15251the mean continuous indoor temperature (Trm) was calculated using hourly temperature data measured by automatic indoor monitoring networks [21]. According to EN15251 indoor thermal environment is divided into categories I, II, and III [24]. Category I is recommended for spaces occupied by the fragile individuals who are highly sensitive to their indoor environment (e.g., children, the elderly, disabled, or sick), category II is should be used for new or remodeled buildings, and category III is the appropriate acceptable level and may be used for existing buildings [25]. EN15251, indoor environmental criteria for the design of residential buildings (bedrooms, drawing room, kitchen, etc.) is based on; summer (0.5 clo) and winter (1.0 clo), and HVAC system design is based on a sedentary level of 1.2 met [23].

### 2.6. Statistical Analysis

We conducted Levene tests to evaluate the homogeneity of variance of all dependent variables (SPSS versions 22; SPSS Inc., Chicago, IL, USA). For all the tests, the results were considered statistically significant when *p* < 0.05. 

Differences between the mean values of the thermal environment (indoor temperature, indoor relative humidity, indoor CO_2_, outdoor temperature, outdoor relative humidity), were compared using a one-way analysis of variance (ANOVA). We compared the differences between rooms (bedroom, living room, kitchen) using the ANOVA mean, and when an overall significant difference was determined, Scheffe’s post-hoc tests were used for one-way ANOVA. The mean of the thermal environment (indoor temperature, indoor relative humidity, indoor CO_2_, outdoor temperature, outdoor relative humidity) when the air conditioner was on versus off during summer and tropical nights was compared using the Student’s *t*-test. ANOVA and Student’s *t*-test were analyzed using SPSS Version 22.0.

The regression model for the indoor and outdoor temperature and relative humidity using R version 3.6.1 (R Foundation for Statistical Computing; Vienna, Austria) was analyzed using the ggpair function (package “GGally”), and the Pearson correlation coefficients (*r*) and *p* values were analyzed with the network correlation matrix (package “qgraph”) using the datasets for each outdoor and indoor space [26,27,28].

## 3. Results

### 3.1. Indoor and Outdoor Thermal Environment Distribution by Season

Table 3 shows the temperature, relative humidity, and CO_2_ for indoors and outdoors from March 2016 to March 2017. It also indicates the thermal environment characteristics with and without air conditioner summer and tropical nights. The results showed that the difference between indoor and outdoor temperatures and relative humidity varied depending on the season. The average indoor and outdoor temperatures for all the seasons were significantly different. During tropical nights the average indoor temperature was 30.6 °C and the average outdoor temperature was 29.7 °C, which was the highest (*p* < 0.001). In contrast, during winter, the average indoor temperature was 20.0 °C, and the average outdoor temperature was 1.4 °C. The average indoor and outdoor relative humidity during tropical nights was 68.2% and 91.6%, respectively, and 45.3% and 60.0%, respectively, during winter. During all seasons, the outdoor relative humidity was higher than the indoor RH (*p* < 0.001). Seasonal CO_2_, comparison results showed that tropical nights were the lowest, with an average of 688 ppm, while winter was the highest, with 1428 ppm, and the average outdoor and indoor CO_2_, concentration was significantly different for all the season (*p* < 0.001).

The indoor temperatures without air conditioning in tropical nights were 30.8 °C, and with air conditioning was 28.5 °C. Our results showed a significant difference between indoor temperatures with air conditioning and without air conditioning (*p* < 0.001). There was also a significant difference between relative humidity without air conditioning and with air conditioning 66.3% and 68.4% respectively, (*p* < 0.001). The outdoor average temperature was 30.3 °C with air conditioning and 29.4 °C without air conditioning. The results showed a significant difference (*p* < 0.001), indicating that outdoor temperatures were higher with air conditioning than without air conditioning.

### 3.2. Correlation Analysis of Indoor and Outdoor Temperature, Relative Humidity, and CO_2_ by Season 

**Figure 1:**
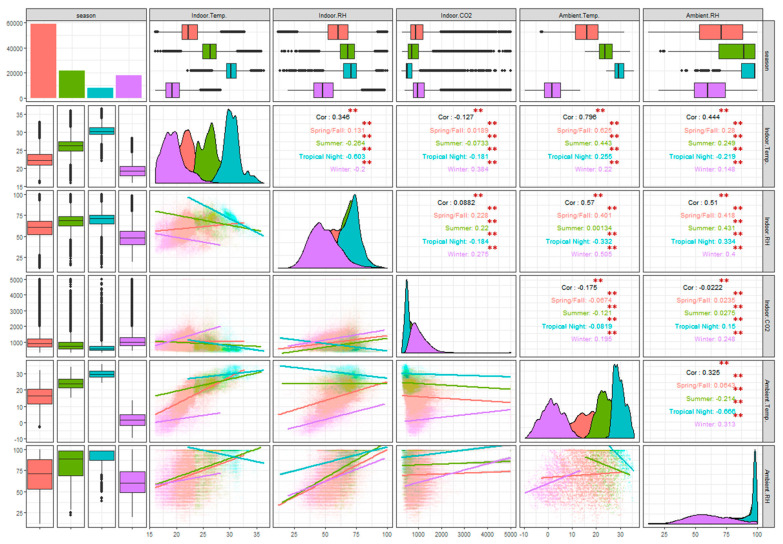
Correlations using scatter plots and regression results for indoor and ambient temperature, relative humidity, and CO_2_ in apartments from March 2016 to March 2017, Seoul, South Korea. ** *p* < 0.05.

Figure 1 shows the average indoor and outdoor temperature and relative humidity for each season with Pearson correlation coefficients (*r*). The outdoor–indoor temperature had a strong correlation (*r* = 0.79, *p* < 0.05) in all seasons. Spring and autumn had the strongest correlation (*r* = 0.62, *p* < 0.05), while summer was slightly weaker than spring and autumn (*r* = 0.44, *p* < 0.05). The outdoor–indoor relative humidity correlation in all the seasons (*r* = 0.51, *p* < 0.05) was weaker than the temperature correlation, and the results of each season analysis showed similar correlations of = (0.41, 0.43, 0.33, and 0.4) in spring–autumn, summer, tropical nights, and winter, respectively (*p* < 0.05). There were significant differences between each season indoor CO_2_–indoor temperature and indoor CO_2_–indoor relative humidity, and consequently low correlation coefficients (=−0.12 and 0.08, respectively; *p* < 0.05).

### 3.3. Comparison of Rooms’ Internal Temperature and Humidity According to External Temperature

**Figure 2:**
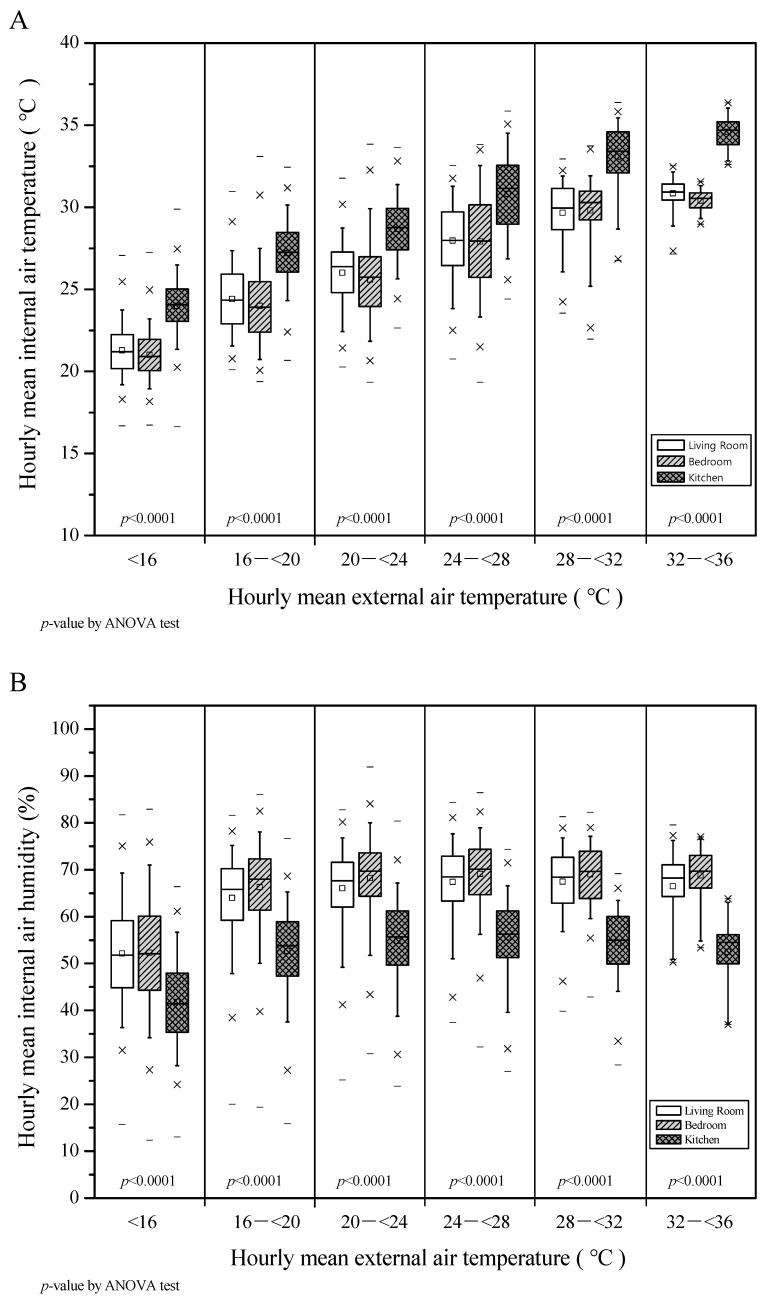
(**A**) Internal vs. external air temperature and (**B**) internal air humidity vs. external air temperature in the eight monitored apartments during a period of one year (Boxplots indicate the minimum, first quartile, median, third quartile, and m max); *p*-value was calculated using a one-way ANOVA test.

Figure 2A,B compare the mean indoor temperature and humidity by space with the mean outdoor temperature divided by intervals. Figure 2A shows that there was a statistically significant difference in the average temperature of each indoor space according to the change in the outdoor temperature. Post-hoc comparisons showed that the kitchen temperature was approximately 2 °C higher than the other spaces. On the other hand, mean indoor temperatures for the living room and bedroom were approximately the same (*p* < 0.001).

Figure 2B analyzed the mean values of indoor relative humidity according to the outdoor temperature changes. The average indoor relative humidity for the spaces/rooms was significantly different (*p* < 0.001). Post-hoc comparisons indicated that the RH of the kitchen was approximately 14% lower than the other spaces, and there was no significant change in seasonal indoor relative humidity.

### 3.4. Distribution of Indoor Thermal Parameters According to ASHRAE Standard 55

**Figure 3:**
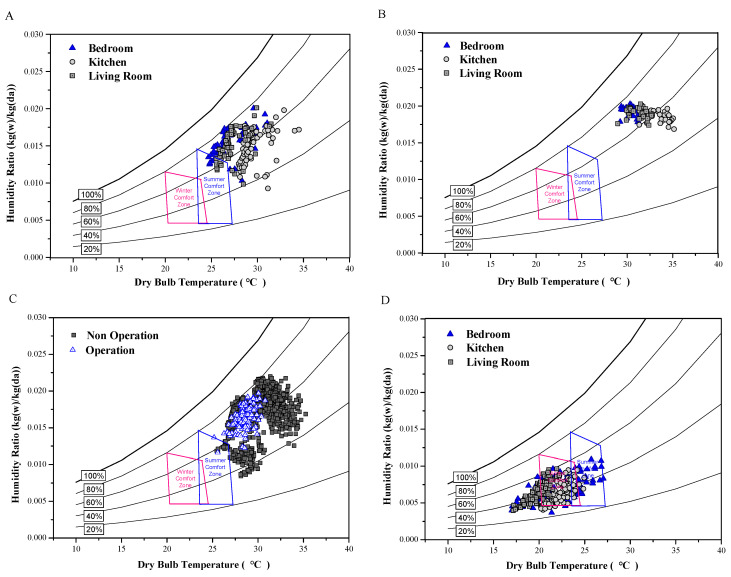
Indoor thermal conditions in the apartments during (**A**) Summer (Jul–Aug, 2016), (**B**) Tropical night in Summer, (**C**) AC was controlled in Summer and Tropical Night at the shows evaluation of thermal living room, and (**D**) Winter (Dec 2016–Feb 2017)**.**

Figure 3 evaluates the thermal comfort by season and space using ASHRAE standard 55. Figure 3A shows the indoor thermal environments during the summer (July–Aug). The results showed that the bedroom and living room were within the same comfort zone, 8.3%, and 10% respectively, while the kitchen fell under a different comfort zone. Temperature ranges observed during a typical Korean summer were mostly higher than the comfort zone. Figure 3B shows the characteristics of indoor thermal environments on tropical nights. The results showed high humidity ratios and high indoor temperatures. Therefore, temperature management is needed, especially for individuals who are vulnerable to high temperatures. Figure 3C compares AC usage. When the AC was on, the indoor temperature was lower than when it was off. However, the indoor thermal environment with air conditioning was not within the comfort zone; therefore, the improvement of summer comfort was not significant. Figure 3D shows the indoor thermal distribution during winter, and these results demonstrate that kitchens, bedrooms, and living rooms, fell within the winter comfort zone, approximately 80%, 57%, and 40 % respectively. 

### 3.5. Adaptive Assessment of Thermal Comfort According to EN 15251

**Figure 4:**
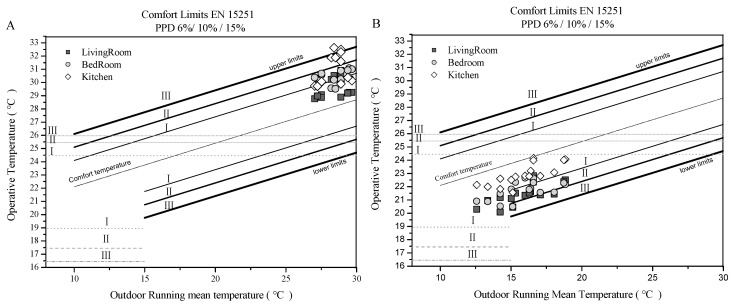
Operative temperatures plotted on the EN 15251 diagram during (**A**) Summer, and (**B**) Autumn (I, II, and III correspond to building categories with different thermal performance requirements).

Figure 4 shows the evaluation of the thermal environment characteristics of each residence using the EN15251 standards. Our findings show that in summer, indoor temperatures are higher than outdoor temperatures. The bedroom and living room summer temperatures fell under category II of EN 15251. However, the temperatures in kitchens during summer were above Category II, which emphasizes the need for thermal environment management. In autumn, the kitchen thermal environment fell under Category II; however, with thermal environments for 25% of living rooms and 12.5 % of bedrooms, above Category II.

### 3.6. Interactions of Thermal Environments for Each Room and Seasons in the Apartments

**Figure 5:**
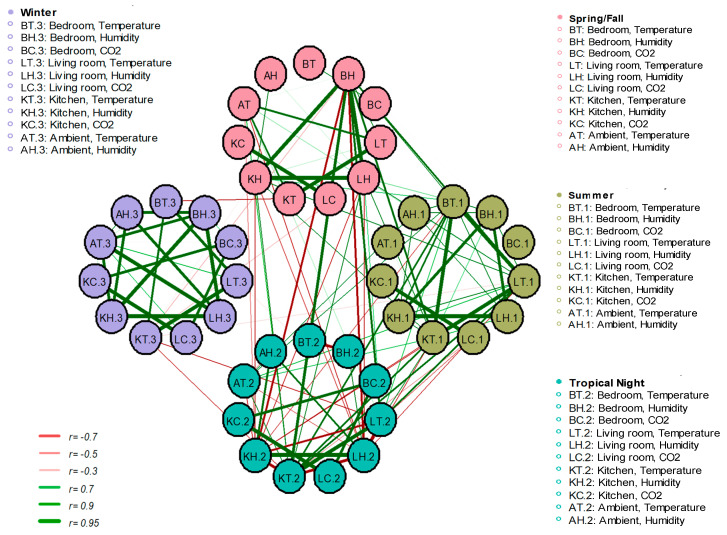
Network correlation analysis of seasonal and spatial thermal environment influencing factors (Pearson correlation coefficients (*r*) were used, and the line indicated *p* < 0.05 to visually distinguish significant correlation factors that were displayed).

Figure 5 shows the overall interaction by means of network correlation analysis of the seasonal and spatial temperature, relative humidity, and CO_2_ for outdoor and indoor, indoor, and indoor relationships. The network correlation lines indicate when *p* < 0.05. The correlations between outdoor–indoor and indoor–indoor spaces were stronger in winter than in other seasons; however, the correlations of winter with other seasons were weak. In contrast, in tropical nights only the living room and the kitchen correlated. The analysis by space showed that the indoor temperature and relative humidity of the living room and kitchen were strongly correlated in all seasons.

The temperatures of the living room and kitchen in spring and autumn showed a strong correlation (*r* = 0.908, *p* < 0.05). The correlation between outdoor–indoor temperature was = (0.785 and 0.741), in the living room and kitchen, respectively. Relative humidity in the bedroom, living room, and the kitchen had a strong correlation (*r* > 0.9, *p* < 0.05). The outdoor–indoor correlation was highest in kitchen (*r* = 0.618, *p* < 0.05).

In summer, the correlation analysis showed that the indoor temperature in the bedroom, living room and kitchen had a strong correlation (*r* > 0.9, *p* < 0.05). In the outdoor–indoor temperature correlation, the kitchens had a weak correlation (*r* = 0.578; *p* < 0.05), unlike the other rooms. Relative humidity in the bedroom, living room and the kitchen had a strong correlation (*r* > 0.88, *p* < 0.05). Relative humidity correlation between outdoor–indoor spaces was weaker than indoor–indoor spaces (*r* > 0.63, *p* < 0.05).

In the tropical nights, the living room and the kitchen the indoor temperature had a strong correlation (*r* = 0.94, *p* < 0.05), and only the kitchen outdoor–indoor temperature correlated (*r* = 0.70, *p*< 0.05). Relative humidity in the living room and kitchen had a strong correlation (*r* = 0.97, *p* < 0.05), and only the living room and the kitchen outdoor–indoor relative humidity correlated = (0.72 and 0.74), respectively (*p* < 0.05).

In winter, there was a strong correlation between indoor–indoor and outdoor–indoor spaces, but the correlation coefficient was slightly lower in outdoor–indoor than in other seasons. Indoor temperature in the bedroom, living room, and kitchen was moderately correlated (*r* > 0.79, *p* < 0.05). Because most spaces are closed up and heated during winter, outdoor–indoor temperature showed a lower correlation than indoor–indoor temperature (*r* > 0.56, *p* < 0.05). A strong correlation was observed between indoor–indoor relative humidity (*r* > 0.91, *p* < 0.05), as well as outdoor–indoor (*r* > 0.82, *p* < 0.05).

Winter showed no correlation with the other seasons. Spring–autumn, summer, and tropical nights were correlated; usually, the temperature was correlated.

### 3.7. Correlation between Indoor Temperature and Relative Humidity, and Human Activities Presence

**Figure 6:**
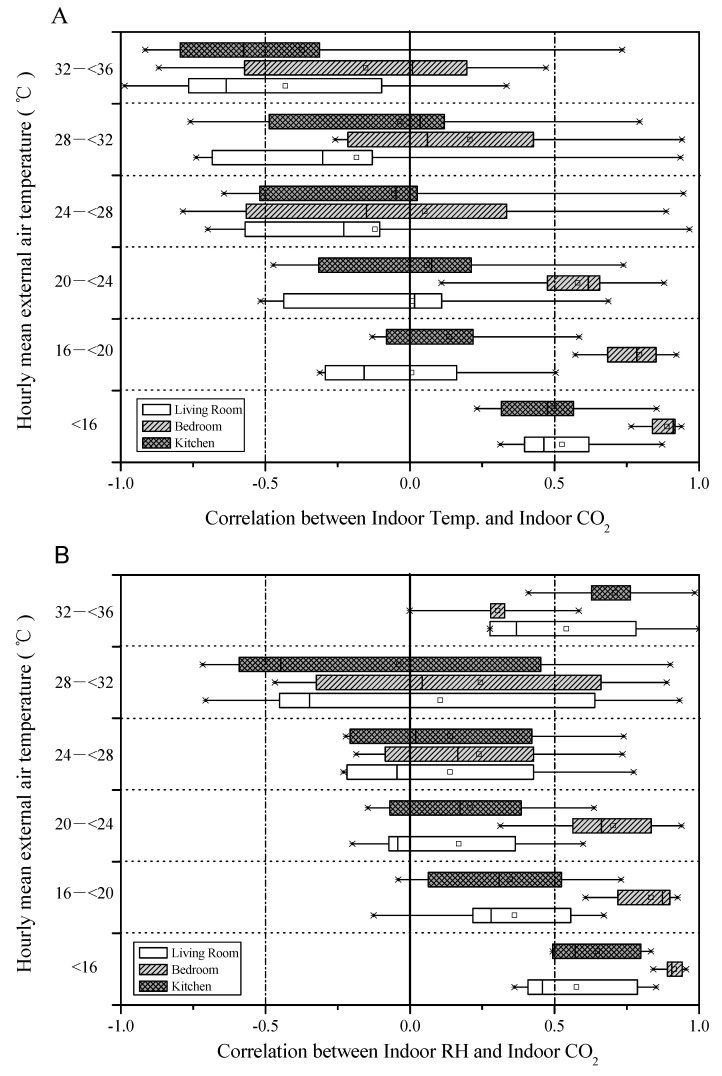
Boxplots of variables correlation in all seasons for the eight apartments (**A**) indoor temperature and carbon dioxide, (**B**) indoor relative humidity and carbon dioxide, from March 2016 to March 2017 (Boxplots indicate the minimum, first quartile, median, third quartile, and max).

Figure 6 analyzes CO_2_ as an indicator of indoor human activities influencing temperature, and relative humidity for each space of the eight households using a Pearson correlation (*r*).

During the heating season (<16 °C), the correlation between indoor CO_2_–indoor temperature and indoor CO_2_–relative humidity was strong. In winter, the correlation between CO_2_ and indoor temperature in the bedrooms was the strongest (*r* = 0.89) because the rooms were mostly closed up and heated at night. The correlation between CO_2_ and indoor relative humidity was *r* = 0.91. During the cooling season (32 °C < 36 °C), the correlation between CO_2_ and indoor temperature exhibited an inverse correlation, but in the relationship between indoor CO_2_ and indoor humidity, the correlation Coefficient increased when the air conditioner was put on.

## 4. Discussion

This is the first study to conduct a long-term analysis of the indoor thermal environment for residential spaces in Seoul, Korea. While there are previous studies on indoor thermal environment, they were often conducted in the living room for only a short period, or covered one climatic season [5,11,17,29].

Using IoT-based real-time wireless sensors, we were able to conduct long-term monitoring and analysis of indoor thermal environment for different spaces; subsequently, our study gave a deep understanding of the indoor thermal environment. We compared the thermal environment variables for each indoor space, our results showed that the kitchen average temperature was approximately 2 °C higher than that of the other spaces, while the average relative humidity was approximately 14% lower. When evaluated using EN15251 model, the summer kitchen was found to go above category II, perhaps because of the heat from the cooking gas that was used in all the households. Similar to the results of our present study, a previous research found the temperature in the kitchen to be approximately 2 °C higher than that of the living room and bedroom, which was attributed to cooking activities [17]. This study suggests that among summer residential spaces in Korea, kitchens have poorest thermal comfort environment.

Without air conditioning, summer and on tropical nights, average temperature was 26.9 and 30.8 °C, respectively. These findings were similar to the results of a previous study on effects of cooling and heating systems on seasonal indoor thermal environment in Korea [29]. The hottest indoor temperature was 36.4 °C; however, with air conditioning in summer and on tropical nights, the indoor temperatures were 25.2 and 28.5 °C, respectively. Previous studies have reported that the use of air conditioners in the residential spaces is limited because of Korea’s industrial-oriented energy policy which led to installation of a progressive electric rates system applied an energy saving measure [29]. For this reason, indoor thermal environment control in summer largely depends on ventilation, a less efficient method. Our present study highlights the need and importance of incorporating air conditioners in managing indoors thermal environment during summer

The average indoor relative humidity in summer and tropical nights was 65.4% and 68.2%, respectively. In winter, the average indoor relative humidity was lower than the outdoor 45.3% and 60.9%, respectively. These results showed that indoors humidity level was low during winter highlighting the need for relative humidity management in winter.

Thermal stress due to extreme thermal environment is a known health risk factor. High temperatures are known to increase respiratory morbidity, and to affect insulin therapy for type 1 diabetes patients [30]. Dry air promotes respiratory infections by reducing the action of cilia, which dry the mucosal surface and remove airway contaminants, before being absorbed by the respiratory mucosa [3,31,32,33]. On the other hand, high-humid environment promote fungal infections, dyspnea, and allergies. However, the standard health-based threshold for indoor temperature and relative humidity in Korea has not been established, and building regulation bodies have not set temperature and humidity thresholds [13,34,35]. The present study reported poor seasonal thermal comfort in regard to temperature and humidity, highlighting the need for establishment of standard threshold for indoor thermal environment.

Long term analysis of indoor thermal environment in this study enabled evaluation of complex contributing factors. We evaluated outdoor–indoor and indoor–indoor correlations for different spaces and seasons. Our results showed a strong correlation between outdoor and indoor dry–bulb temperature (*r* = 0.79) as well as a strong correlation for indoor–indoor spaces temperature.

For relative humidity, the correlation analysis of the total time average showed that the outdoor–indoor correlation (*r* = 0.51) was weaker than that of the temperature. The network correlation analysis by season and space showed that the correlation between indoor–indoor was strong, while in winter, the correlation between outdoor–indoor was strong. These results support a previous study that reported that the correlation analysis using absolute humidity (AH) can be more accurate than relative humidity, because outdoor relative humidity is a poor indicator of indoor relative humidity [3,11]. In addition, the correlation analysis with CO_2_ as a representative indicator of human activity showed that relative humidity and CO_2_ had a strong correlation in winter. These results are in agreement with a previous study that reported that relative humidity is influenced by indoor human activities at a greater extent than outdoor human activities. The study also reported that various human activities, such as respiration, pets rearing, showering, cooking, dish washing, and cleaning, affect indoor relative humidity [36].

Assessments of personal exposure to thermal environmental risks are a key factor to manage of the indoor thermal environment. The first step in evaluating indoor personal exposure is to select an on-site measurement and analysis method. In this study, field measurements using IoT-based real-time wireless sensors were conducted in selected apartment kitchen, living room, and bedroom spaces for a year. The miniaturization of IoT monitoring devices and the hosting of data on cloud servers using Wi-Fi networks have expanded the applicability of field measurements. Interestingly our present study measurement results were similar to those of the previous studies, which means that this technique can adequately replace the existing use of expensive measurement equipment. Long-term monitoring and analysis in this study was effective in evaluating the complex indoor thermal environments because this approach allowed evaluation of thermal environment characteristics for each climatic seasons and different residential spaces. Therefore, we are confident that the with large scale data accumulated through IoT-based monitoring devices it is possible to provide innovative information, such as evaluation, predictions, guidelines, and solutions for the thermal environment in the near future.

However, our study had several limitations. This study focused on a small geographic area and a small number of apartments. Therefore, our results might not give an accurate picture since the temperature and relative humidity may differ, depending on the type of housing and the region (suburb, rural). Further studies including other types of environments, such as work environments, office buildings, and nursing homes are required. Behavioral information was not collected on a daily basis; therefore, personal exposure cannot be accurately linked to the time spent in the house, activity patterns, and movements. These results provide insight into thermal environment effects on health.

## 5. Conclusions

To derive an accurate method to measure and analyze indoor thermal environmental data viable for application in healthcare, we did long-term monitoring and analysis of thermal environmental using IoT-based real-time wireless sensors. We monitored the climate and indoor thermal environment characteristics of different residential spaces (bedroom, living room, and kitchen) for one year (covering all climatic seasons). Based on our results and consistency of our findings with similar studies, our method proved efficient. Our present study also proved IoT-based monitoring devices as a potential approach for evaluating personal exposure to indoor thermal environmental risks. Our results confirmed that the outdoor temperature was an important influencing factor for the indoor thermal environment, while the indoor relative humidity is a good indicator of residential space occupant’s lifestyle. However, indoor temperature and relative humidity are affected by season and room space; therefore, network correlation analysis of variables is important in evaluating health effects of thermal environment through all climatic seasons. Our findings highlighted the need for temperature management in summer, RH management in winter and kitchen thermal environment management during summer and tropical nights.

## Figures and Tables

**Table 1:** Case study information on the dwellings.

Code	Built In	Apartment Area (m^2^)	Number of Bedrooms	Floor Level	House Type	Ventilation
A	2002	109	3	2	Apartment (Concrete)	NV * + AC **
B	2001	82	2	8	Apartment (Concrete)	NV * + AC **
C	1997	82	2	15	Apartment (Concrete)	NV * + AC **
D	2010	109	3	10	Apartment (Concrete)	NV * + AC **
E	2005	107	3	11	Apartment (Concrete)	NV * + AC **
F	2000	82	2	20	Apartment (Concrete)	NV * + AC **
G	1992	93	3	18	Apartment (Concrete)	NV * + AC **
H	2001	82	2	8	Apartment (Concrete)	NV * + AC **

* NV = Natural Ventilation. ** AC = Air Conditioning.

**Table 2:** Type and characteristics of the measuring devices.

Parameter	Measuring Device	Measurement Range	Resolution	Accuracy
Temperature	AQ-0115V (Coway Co. Ltd., Korea)	(−20 to 45) °C	0.1 °C	±0.5 °C
Relative humidity		(5 to 95) % RH	0.1 % RH	±0.1% RH
Globe temperature	Testo 480 (Testo Co. Ltd., Germany)	(0 to 120) °C	0.1 °C	±0.5 °C (0 to 50 °C) ±1 °C (50 to 120 °C)
Airflow rate	WGT-10 (Hario SCI, Japan)	(0.05 to 20) m/s	0.01 m/s	±0.03 m/s

**Table 3:** Seasonal distribution of hourly average indoor and ambient temperature, humidity, and CO_2_ in apartments.

Type		Indoor						Ambient			
		Temp. (°C)		Relative Humidity (%)		CO_2_ (ppm)		Temp. (°C)		Relative Humidity (%)	
		Mean ± S.D.	Min–Max	Mean ± S.D.	Min–Max	Mean ± S.D.	Min–Max	Mean ± S.D.	Min–Max	Mean ± S.D.	Min–Max
Spring/Fall ^1^		23.0 ± 2.5	16.1–32.7	57.1 ± 11.0	12.4–83.7	997.6 ± 497.4	304.8–5000	15.5 ± 6.8	−3.0–31.8	70.2 ± 21.0	12.0–98.0
Summer	Total ^1^	26.6 ± 2.0	22.6–35.5	65.4 ± 8.9	26.1–83.9	827.8 ± 349.5	305.2–3398	23.6 ± 3.5	15.2–34.0	82.5 ± 18.5	22.0–98.0
	AC on ^2^	25.2 ± 0.6	24.9–25.5	68.3 ± 1.3	66.5–69.4	962.3 ± 29.0	924.1–994.2	25.9 ± 1.3	25.3–27.1	87.1 ± 9.3	79.0–98.0
	AC off ^2^	26.9 ± 2.0	22.6–35.5	65.4 ± 8.9	26.1–83.9	815.8 ± 349.6	305.2–3398.0	23.3 ± 3.5	15.2–34.0	82.5 ± 18.5	22.0–98.0
Tropical Night	Total ^3^	30.6 ± 1.7	24.6–36.4	68.2 ± 7.7	37.0–84.4	688.3 ± 430.7	331.7–4372.0	29.7 ± 2.4	24.2–35.7	91.6 ± 10.2	39.0–98.0
	AC on ^3^	28.5 ± 1.1	24.6–31.2	66.3 ± 5.7	46.8–81.2	1424.5 ± 641	465.2–4369.8	30.3 ± 1.7	27.1–34.6	93.4 ± 9.5	55.0–98.0
	AC off ^3^	30.8 ± 1.6	26.1–36.4	68.4 ± 7.8	37.0–84.4	631.5 ± 350.8	331.7–4372.0	29.4 ± 2.4	24.2–35.7	91.5 ± 10.3	39.0–98.0
Winter ^1^		20.0 ± 2.1	16.0–28.3	45.3 ± 9.0	18.1–73.3	1428.6 ± 642	489.3–4325.4	1.4 ± 4.5	−9.6–13.4	60.9 ± 16.5	20.0–98.0
^1^*p*-value by ANOVA		*p* < 0.001		*p* < 0.001		*p* < 0.001		*p* < 0.001		*p* < 0.001	
^2^*p*-value by T-test		*p =* 0.16		*p =* 0.66		*p =* 0.85		*p* < 0.001		*p* < 0.001	
^3^*p*-value by T-test		*p* < 0.001		*p* < 0.001		*p* < 0.001		*p* < 0.001		*p* < 0.001	

^1^ Mean values for each season compared using a one-way ANOVA test. ^2^ The mean difference for temperature and relative humidity with and without air conditioning tested with a Student’s *t*-test in summer. ^3^ The mean difference for temperature and relative humidity with and without air conditioning tested with a Student’s *t*-test on tropical nights.
